# Ascorbate deficiency increases progression of shigellosis in guinea pigs and mice infection models

**DOI:** 10.1080/19490976.2023.2271597

**Published:** 2023-10-24

**Authors:** Jurate Skerniskyte, Céline Mulet, Antonin C. André, Mark C. Anderson, Louise Injarabian, Achim Buck, Verena M. Prade, Philippe J. Sansonetti, Sophie Reibel-Foisset, Axel K. Walch, Michel Lebel, Jens Lykkesfeldt, Benoit S. Marteyn

**Affiliations:** a Institut de Biologie Moléculaire et Cellulaire, Architecture et Réactivité de l’ARN, Université de Strasbourg, Strasbourg, France; b Unité de Pathogénie Microbienne Moléculaire, Institut Pasteur, Université de Paris, Paris, France; c Research Unit Analytical Pathology, Helmholtz Zentrum München – German Research Center for Environmental Health, Neuherberg, Germany; d Collège de France, Paris, France; e Chronobiotron, CNRS, Université Strasbourg, Strasbourg, France; f Centre de recherche du CHU de Québec, Faculty of Medicine, Université Laval, Québec, Canada; g Section for Experimental Animal Models, Faculty of Health and Medical Sciences, University Copenhagen, Copenhagen, Denmark; h Unité de Pathogenèse des Infections Vasculaires, Institut Pasteur, INSERM U1225, Paris, France; i University of Strasbourg Institute for Advanced Study (USIAS), Strasbourg, France

**Keywords:** *Shigella*, shigellosis, intestinal infection, infection animal model, ascorbate deficiency, Animal models of GI infection or GI-diseases with microbial components.

## Abstract

*Shigella* spp. are the causative agents of bacterial dysentery and shigellosis, mainly in children living in developing countries. The study of *Shigella* entire life cycle *in vivo* and the evaluation of vaccine candidates’ protective efficacy have been hampered by the lack of a suitable animal model of infection. None of the studies evaluated so far (rabbit, guinea pig, mouse) allowed the recapitulation of full shigellosis symptoms upon *Shigella* oral challenge. Historical reports have suggested that dysentery and scurvy are both metabolic diseases associated with ascorbate deficiency. Mammals, which are susceptible to *Shigella* infection (humans, non-human primates and guinea pigs) are among the few species unable to synthesize ascorbate. We optimized a low-ascorbate diet to induce moderate ascorbate deficiency, but not scurvy, in guinea pigs to investigate whether poor vitamin C status increases the progression of shigellosis. Moderate ascorbate deficiency increased shigellosis symptom severity during an extended period of time (up to 48 h) in all strains tested (*Shigella sonnei*, *Shigella flexneri* 5a, and 2a). At late time points, an important influx of neutrophils was observed both within the disrupted colonic mucosa and in the luminal compartment, although *Shigella* was able to disseminate deep into the organ to reach the sub-mucosal layer and the bloodstream. Moreover, we found that ascorbate deficiency also increased *Shigella* penetration into the colon epithelium layer in a Gulo^−/−^ mouse infection model. The use of these new rodent models of shigellosis opens new doors for the study of both *Shigella* infection strategies and immune responses to *Shigella* infection.

## Introduction

Bacillary dysentery or shigellosis remains a major disease burden especially in developing countries; in 2010 annual shigellosis mortality was estimated to 123,000 deaths worldwide among 88.4 million cases, mainly among children under the age of five.^1^ In 2017, *Shigella* was included in the WHO list of the 12 antibiotic-resistant «priority pathogens» that pose the greatest threat to human health.^2^ Shigellosis is associated with fever, abdominal cramps and rectal inflammation. Dysenteric stools characteristically contain erythrocytes, polymorphonuclear neutrophils (PMNs) and mucus. Shigellosis is characterized by the specific invasion and destruction of the human colonic mucosa by *Shigella* spp., which are transmitted via the feco-oral route. To date, no animal reservoirs have been reported. Although the etiologic agent, *Shigella* spp., has been identified during the last century, shigellosis represents a major threat to public health since no licensed vaccine is available.^3^

The main risk factors of shigellosis are poor sanitation and limited access to clean water.^4^ Malnutrition has also been shown to correlate with an increased risk of shigellosis.^5,6^ It has been shown that Vitamin A^7^ or zinc supplementation^8^ reduce the severity of acute shigellosis in malnourished children in Bangladesh and has been recommended by the WHO as supportive care in association with antimicrobials.^9^

In addition to these prophylactic treatments, the development of a pan-*Shigella* vaccine remains urgently needed. The lack of a suitable animal model of shigellosis, which recapitulates the entire disease process from an oral challenge to the specific infection and destruction of the colonic mucosa, represents a major roadblock for the development of safe and efficacious vaccine candidates, but also to fully understand the *Shigella* virulence cycle. Over the past few decades, several animal models of shigellosis have been proposed, including monkeys, rabbits, mice, and guinea pigs. All of them have their own limitations, while none of them recapitulates all shigellosis symptoms.^2^ The infection of the colonic mucosa by *Shigella* following an oral challenge was reported in rhesus macaques (*Macaca mulatta*).^10^ Rare occurrence of bacteria in the lamina propria was observed in zinc-deficient mice.^11^ Clinical features of shigellosis were observed in orally infected NAIP – NLRC4-deficient mice, however shigellosis symptoms remain transient and animal recovery occurred after a few days.^12^ A transient colonic infection has been reported in young guinea pigs (Hartley strain) upon intrarectal inoculation.^13^ In other proposed models, including the sereny test (guinea pig), the ileal loop model (rabbit) or the intravenous or intranasal infections (mouse), the targeted organs are different, making it difficult to interpret the results. It is important to highlight that conventional mice are not susceptible to *Shigella* infection for reasons that remain unclear.

A potent humoral immunological response to *Shigella* infection (including IgA and IgG secretion) has been reported in shigellosis patients and has been observed in several animal models (monkeys, mice, rabbit, guinea pigs).^14^ It has been reported to be serotype-specific and mainly directed toward *Shigella* lipopolysaccharide (LPS-associated O-antigen repeats).^15^ However, no direct correlation between the induced immune response and potential protection against shigellosis has been confirmed. Consequently, vaccine candidates are currently evaluated in clinical trials (phases I-III) only based on their immunogenicity (assessed in preclinical models, mainly mice), with no proof of protection efficacy. This strategy is time-consuming, risky, expensive, and should be optimized to improve the chances of success; this includes the validation of a suitable animal model of shigellosis.^16^

In this study, we aimed to identify a novel susceptibility factor for shigellosis to develop and validate new animal models of shigellosis, that would faithfully reproduce all physiopathologal and clinical symptoms. We hypothesized that ascorbate deficiency may be an unknown susceptibility risk factor for shigellosis based on physiological reasons and historical reports. First, acute shigellosis has only been reported in humans, non-human primates, and guinea pigs, which are among the few mammalian species unable to synthesize ascorbate, due to the lack of a functional L-gulonolactone oxidase enzyme (GULO), which converts L-gulono-1,4-lactone to L-ascorbic acid (ascorbate or Vitamin C). Conversely, conventional mice express functional GULO and synthesize ascorbate. Mice are not susceptible to *Shigella* infection, regardless of the infection route,^17^ suggesting that optimal ascorbate supply may limit shigellosis severity. Honjo *et al*. showed that shigellosis symptoms are more severe in ascorbate-deficient monkeys (cynomolgus monkeys) upon oral challenge, suggesting a possible link between ascorbate status and disease susceptibility.^18^

Second, the association of scurvy (caused by severe and prolonged ascorbate deficiency) and dysentery was first reported by Lind in 1753.^19^ Since then, it was hypothesized that both scurvy and chronic diarrhea may be nutritional deficiency syndromes, affecting populations suffering from malnutrition (ie. soldiers, seamen).^20^ Diarrhea was considered as a “symptom” or “part” of scurvy and characterized as “scorbutic fluxes” or “scorbutic diarrhea”. Importantly, ascorbate supplementation allowed the treatment of both scurvy and diarrhea symptoms.^19^ Severe ascorbate deficiency has been confirmed as the main cause of scurvy. However, no other clinical symptoms or diseases have been associated with moderate ascorbate deficiency. Our recent study demonstrated that infants with hypovitaminosis C are at a significantly higher risk of bacterial infectious diseases, including shigellosis.^21^

In this study, we developed and validated an ascorbate-deficient guinea pig model of shigellosis, which recapitulates shigellosis symptoms upon oral challenge with *S. flexneri* 2a and 5a and *S. sonnei*. We showed that *Shigella* penetration into the colon mucosa was also increased in ascorbate-deficient Gulo^−/−^ mice compared to conventional mice. Taken together, these results demonstrate that ascorbate deficiency accelerates the progression of shigellosis symptoms and severity in these infection models.

## Results

### Optimization of the guinea pig diet to induce a moderate ascorbate deficiency without scurvy symptom

The guinea pig model of shigellosis reporting the colonization and destruction of the colonic mucosa by *Shigella* was described by Shim and colleagues^13^ and consists of an intrarectal challenge of young guinea pigs (2-week) with *S. flexneri* 2a and *S. flexneri* 5a. It was reported that severe and acute colitis was induced 8 h post-infection (p.i.), and that symptoms disappeared by 24 h-48 h p.i., thus representing an important limitation of the model. In addition, it was shown that older animals (5-week and more) are no longer susceptible to *Shigella* infection.^13^ We confirmed these results in our laboratory and used this model in routine studies to characterize *Shigella* virulence mechanisms *in vivo*.^22–24^

**Figure 1.**
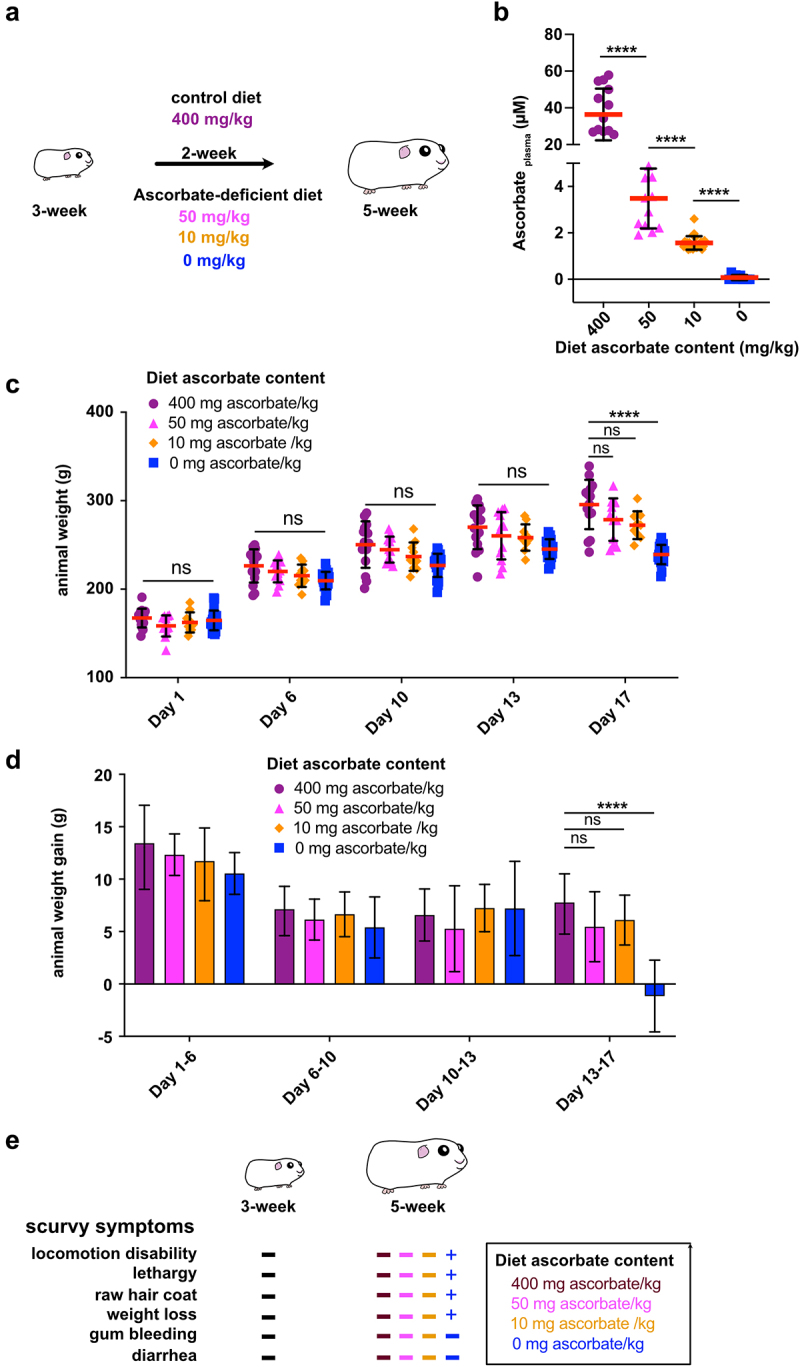
Optimization of diet ascorbate content to induce guinea pig moderate ascorbate deficiency. (a) Young guinea pigs (3-week) were fed for 2 weeks with diets containing various ascorbate concentration to induce moderate ascorbate deficiency without inducing scurvy, caused by severe ascorbate deficiency. Commercial diet contains 400 mg ascorbate/kg (Safe); suboptimal ascorbate supply was obtained with diets containing 50 mg, 10 mg and 0 mg ascorbate/kg. (c) Guinea pig ascorbate deficiency was confirmed by dosing plasma ascorbate concentration. Results are expressed as mean ± S.D., **** indicates t-test *p* < .0001 (*n* > 10 animals per group). (c-d) Moderate ascorbate deficiency induction did not lead to a significant reduction of weight gain, animals were weighted regularly (day 1, 6, 10, 13 and 17) during this period of time. Results are expressed as mean ± S.D., ‘ns’ indicates t-test *p* > .05, **** indicates *p* < 0.0001 (*n* > 10 animals per group). (e) the absence of scurvy symptoms (as indicated) was confirmed in young animals (3-week) and after 2-week feeding with diets containing 400, 50, 10 and 0 mg ascorbate/kg. ‘+’ indicates the corresponding scurvy symptom has been observed in at least one animal of the group.

In this study, we aimed to evaluate the impact of moderate ascorbate deficiency on the severity of shigellosis in guinea pigs. Animals were fed with specific diets containing sub-optimal ascorbate amounts for 2 weeks (Figure 1a). The amount of ascorbate in the diet allowing for an optimal supply to guinea pigs was 400 mg ascorbate/kg (see Methods). We evaluated the impact of diets containing 50, 10, and 0 mg ascorbate/kg (Figure 1a) on guinea pig plasma ascorbate concentration (Figure 1b) and animal weight (Figure 1c,d) and reported the absence of scurvy symptoms (Figure 1e).

The guinea pig plasma ascorbate concentration upon optimal ascorbate supply was 36.4 ± 14.1 µM, confirming results from other studies,^25^ which is in the same range although lower compared to human plasma ascorbate concentration (49.5 ± 14.2 µM).^26^ When guinea pig diet was devoid of ascorbate (0 mg/kg), the plasma ascorbate concentration was 0.1 ± 0.1 µM, as anticipated, confirming that a 2-week diet was sufficient to induce deficiency. A moderate ascorbate deficiency was induced when guinea pigs were fed a diet containing 50 mg ascorbate/kg (3.5 ± 1.3 µM) or 10 mg/kg (1.6 ± 0.3 µM) (Figure 1b). We further aimed to define the most suitable low-ascorbate diet that ensured a significant reduction in guinea pig plasma ascorbate concentration while preventing scurvy.

The animal weight of all groups increased for 13 days and was not significantly different at any time point (Figure 1c,d). However, between days 13 and 17, significant weight loss was observed in the absence of ascorbate (Figure 1d, 0 mg ascorbate/kg, *p* < .001), but not in diets containing sub-optimal ascorbate amounts (Figure 1d). In addition, no scurvy symptoms (locomotion disability, lethargy, raw hair coat, or weight loss) were observed in animals fed this diet, as opposed to those devoid of ascorbate (Figure 1e).

In conclusion, a diet containing 10 mg ascorbate/kg induced a significant decrease in plasma ascorbate concentration (Figure 1b), without affecting animal growth (Figure 1c,d) or causing scurvy (Figure 1e), and was further used in this study.

### Moderate ascorbate deficiency increases shigellosis severity upon shigella intrarectal challenge

In humans, shigellosis symptoms appear between 24 h to 48 h p.i. As mentioned above, guinea pigs fed an optimal diet did not develop such long-term infectious processes (*Shigella* was cleared by the immune system 24 h p.i.). In addition, only young guinea pigs (2-week, just after weaning) were susceptible to *Shigella* infection inoculated intrarectally at high-dose: older animals (5-week or more) were not.^13^ An oral *Shigella* challenge did not lead to infection and destruction of the colonic mucosa, as observed in humans (transmission through the fecal-oral route).

**Figure 2.**
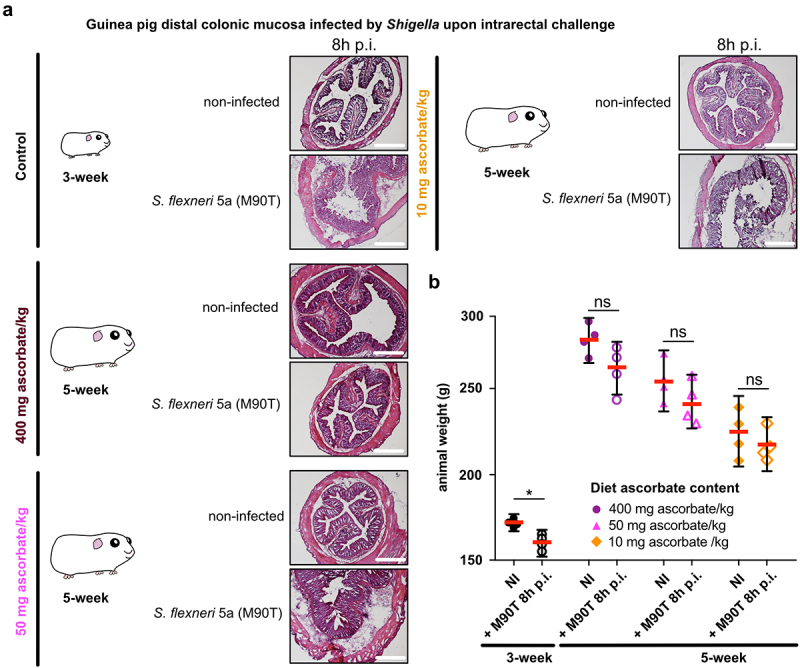
Moderate ascorbate deficiency increases the susceptibility of adult guinea pigs to *Shigella* infection upon intrarectal challenge. (a) Young (3-week) and older guinea pigs fed with 400, 50 and 10 mg ascorbate/kg diets (5-week) were challenged intrarectally with 10^10^ CFU *S. flexneri* 5a (M90T). 8 h p.I. animals were sacrificed, and distal colonic samples were collected, stained with haematoxylin eosin and compared to non-infected tissues. Scale bars are 30 mm. (b) Animal weight was measured before and after infection. Results are expressed as mean ± S.D., * indicates t-test *p* < .05. Representative images are presented of at least 5 slices from 3 different animals.

Using *S. flexneri* 5a as a model, we first confirmed that the colonic mucosa was disrupted 8 h p.i. by *Shigella* upon intrarectal challenge in young animals (3-week) fed a high-ascorbate diet (400 mg ascorbate/kg) (Figure 2a). We confirmed that older animals (5-week) fed a similar diet were not susceptible to *Shigella* infection. Conversely, we observed that ascorbate-deficient guinea pigs (5-week) remained susceptible to *S. flexneri* 5a infection (colonic mucosa destruction, leukocyte infiltration, edema), and this phenotype was observed in animals fed both low-ascorbate diets (50 and 10 mg ascorbate/kg) (Figure 2). However, no significant weight loss was observed at 8 h p.i. in 5-week guinea pigs (due to high variability of the results), as observed in young animals (Figure 2b, *p* < .05) and previously reported.^13^

We hypothesized that weight loss might be observed in 5-week guinea pigs during long-term infection. To address this question, the *Shigella* infection course was studied in ascorbate-deficient guinea pigs over longer infection periods using various *Shigella* species.

### *Prolonged infection processes are induced by* S. flexneri *5a,*S. flexneri *2a and* S. sonnei *in ascorbate-deficient guinea pigs*

**Figure 3.**
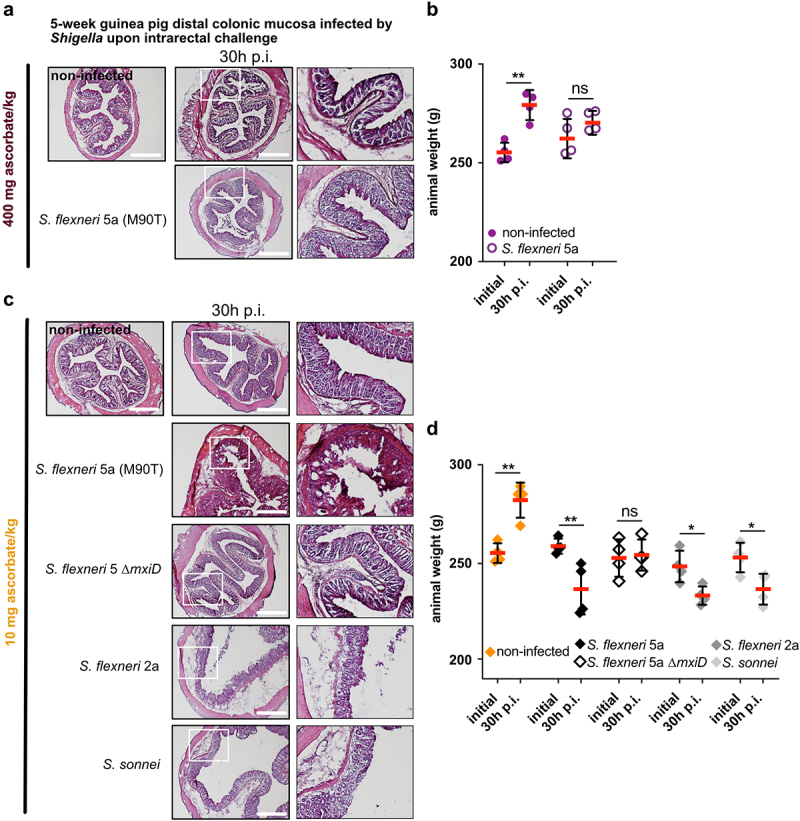
*Shigella* spp. cause destruction of the colonic mucosa and induce weight loss in ascorbate-deficient guinea pigs for extended period of time. (a) 5 weeks old guinea pigs (400 mg ascorbate/kg) and (c) ascorbate-deficient guinea pigs (10 mg ascorbate/kg diet) were infected intrarectally with *S. flexneri* 5a (wt and ∆*mxiD* strains), *S. flexneri* 2a and *S. sonnei*. 30 h p.I. animals were sacrificed, and distal colonic samples were collected, stained with haematoxylin eosin and compared to non-infected tissues. Scale bars are 30 mm. On the right panel, x3 magnification of white squares (left panel) are shown. (b and d) Animals were weighted before infection and 30 h p.I. Results are expressed as mean ± S.D., ‘ns’ indicates t-test *p* > .05, * indicates *p* < .05, ** indicates *p* < .01 (4 animals per group). Representative images are presented of at least 5 slices from 3 different animals.

We evaluated the severity of *Shigella* infection 30 h p.i. upon intrarectal infection in 5-week ascorbate-deficient guinea pigs compared to non-infected controls. As previously reported,^13^ we did not observe severe symptoms of shigellosis 30 h p.i.in guinea pigs fed with a 400 mg ascorbate/kg diet (Figure 3a). Infected animals kept gaining weight during the infection period (30 h, *p* > .05), and to a lesser extent than non-infected animals (Figure 3b), confirming that a transitory infection occurred at an early time point, *e.g*. 8 h p.i. (Figure 2).

Conversely, severe *Shigella* infections were observed 30 h p.i. (intrarectal challenge) in 5-week ascorbate-deficient guinea pigs (colonic mucosa destruction, leukocyte infiltration, edema), using *S. flexneri* 5a, *S. flexneri* 2a, or *S. sonnei*, but not with an avirulent plasmid-cured *S. flexneri* 5a ∆*mxiD* mutant (Figure 3c). If non-infected guinea pigs gained weight during this period, a significant weight loss was induced by *Shigella* infection in all groups, but not with *S. flexneri* 5a ∆*mxiD* (Figure 3d).

These results demonstrate that ascorbate deficiency increases the severity of *Shigella* spp. infection in guinea pigs. We previously reported that *Shigella* infection induced a significant decrease in human plasma ascorbate concentration,^26^ through a mechanism that remains to be defined. Here, we further investigated in a complementary approach the abundance of ascorbate within different colonic mucosa layers and its modulation during *Shigella* infection.

### Shigella infection induces a systemic ascorbate concentration decrease and occurs in low-ascorbate microenvironments

**Figure 4.**
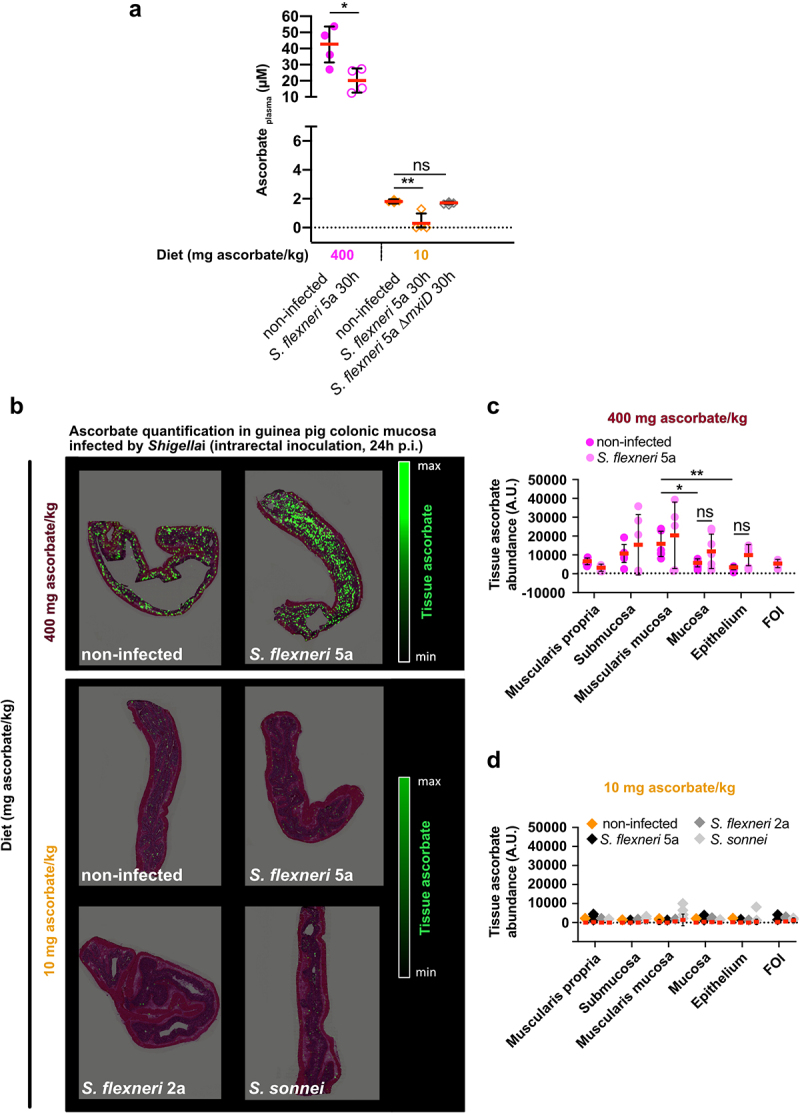
Ascorbate deficiency is associated with reduction of ascorbate in plasma and within colonic mucosa layers. (a) Ascorbate concentration in plasma was measured by HPLC and expressed as mean ± S.D., * indicates t-test *p* < .05, ** indicates *p* < .01. (b) Ascorbate abundance was quantified within the different layers of the colonic mucosa by imaging mass spectrometry in tissues from animals fed with 400 mg ascorbate/kg and 10 mg ascorbate/kg diets, infected or not with indicated strains for 30 h (intrarectal challenge). (c-d) Quantification in different layers are averaged from three areas designed on three independent samples and expressed as mean ± S.D., * indicates t-test *p* < .05, ** indicates *p* < .01, FOI – foci of infection. Representative images are presented of at least 5 slices from 3 different animals.

First, we confirmed that in control animals (fed a high-ascorbate diet) a decrease in the plasma ascorbate concentration upon *S. flexneri* 5a infection (30 h p.i., intrarectal challenge) (Figure 4a). A significant decrease was observed in ascorbate-deficient guinea pigs upon *S. flexneri* 5a infection (30 h p.i., intrarectal challenge), but not in *S. flexneri* 5a ∆*mxiD* mutant, despite the fact that the plasma ascorbate concentration was already low under basal conditions.

The determination of local ascorbate abundance remains difficult, and only a few quantitative data are available in the colon.^27^ Ascorbate abundance was further quantified within the colonic mucosa layers using mass imaging spectrometry^28,29^ in the same models of *Shigella* infection (Figure 4b). We first observed that under basal conditions, the ascorbate distribution was heterogeneous between layers. In control animals (fed with high-ascorbate diet), higher amounts of ascorbate were detected within the submucosa and muscularis mucosa than in the mucosa or epithelium (Figure 4c). In ascorbate-deficient guinea pigs, ascorbate was almost undetectable in any layer under basal conditions (Figure 4d), showing for the first time that ascorbate deficiency is induced, and not only the plasma ascorbate concentration but also its local distribution within the colon is strongly decreased. No effect of *Shigella* infection on ascorbate abundance within the colonic layers was observed in control or ascorbate-deficient animals. However, ascorbate was not detected within foci of infection in either control or ascorbate-deficient animals (Figure 4(c,d)). Since *Shigella* colonies specifically the colonic mucosa, the distribution of ascorbate with the small intestine has not been examined in this study. Further investigations will be required to address this point, since ascorbate is absorbed in this section of the gastrointestinal tract.

As we confirmed that ascorbate deficiency increased shigellosis symptom severity, we further aimed to evaluate whether the overall life cycle of *Shigella*, from an oral challenge to colon colonization, could be recapitulated in this new shigellosis animal model during extended periods of infection.

### Efficient colonization of the colonic mucosa by shigella is observed in ascorbate-deficient guinea pigs

**Figure 5.**
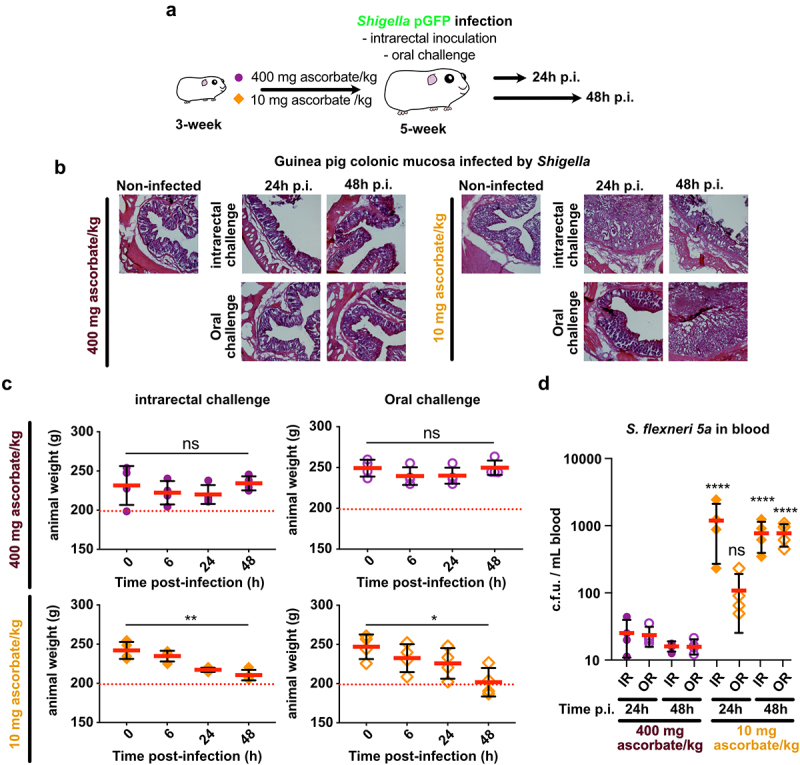
Ascorbate-deficient guinea pigs are susceptible to *Shigella* infection, upon intrarectal or oral challenge, over extended periods of time. (a) ascorbate-deficient guinea pigs (10 mg ascorbate/kg diet) and control animals (400 mg ascorbate/kg diet) were infected intrarectally or orally with 10^10^ CFU *S. flexneri* 5a. 24 h or 48 h p.I. (b) animals were sacrificed, and distal colonic samples were collected, stained with haematoxylin eosin and compared to non-infected tissues. (c) weight loss in guinea pigs infected by intrarectal and oral challenge is expressed as mean ± S.D., * indicates t-test *p* < .05, ** indicates *p* < .01. (d) *S. flexneri* 5a potential translocation to the bloodstream was assessed by plating blood samples on TSA culture plates. Results are expressed as mean ± S.D., ‘ns’ indicates t-test *p* > .05, **** indicates *p* < .0001 (4 animals per group). Representative images are presented of at least 5 slices from 3 different animals.

**Figure 6.**
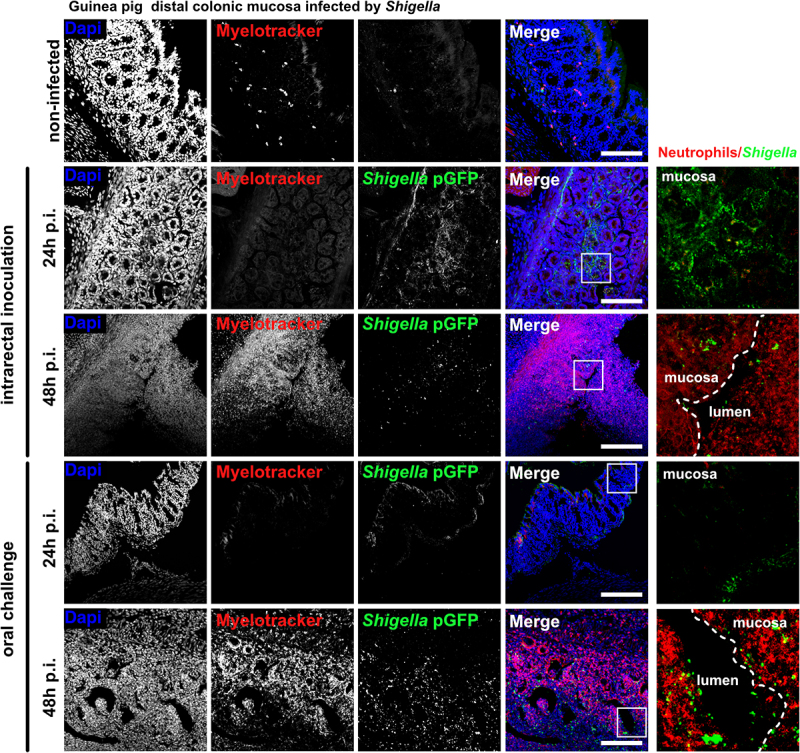
*S. flexneri* 5a colonize and attract neutrophils into infection foci in ascorbate-deficient guinea pigs. *S. flexneri* 5a pGFP (green) and neutrophils (red) were detected by immunofluorescence within the distal colonic mucosa of ascorbate-deficient guinea pigs (10 mg ascorbate/kg diet) challenged orally or intrarectally for 24 and 48 h with 10^10^ CFU. Nuclei were stained with DAPI (blue), neutrophils were stained with myelotracker-Cy3 (red). Scale bars are 150 µm. On the right panel, x5 magnification of merged images (white squares) are shown : *S. flexneri* 5a (green) and neutrophils (red). Detection of *S. flexneri* pGFP and neutrophils in tissues from control animals (400 mg ascorbate/kg diet) is shown in fig. S1. Representative images are presented of at least 5 slices from 3 different animals.

To recapitulate *Shigella* life cycle, control and ascorbate-deficient guinea pigs were challenged orally with *S. flexneri* 5a pGFP and long-term infections (24 h and 48 h) were performed (Figure 5(a)), animals were weighted and colonic mucosa colonization and destruction by *Shigella* were then evaluated (Figure 5b). As a major sign of shigellosis severity, we found that ascorbate-deficient animal weight continuously decreased during the time of infection, reaching 20% weight-loss 48 h p.i. upon oral and intrarectal challenge (Figure 5c); this phenotype was not observed in control animals. We observed comparable inflammation and infection symptoms within the colonic mucosa at 24 h and 48 h p.i. upon intrarectal and oral challenge major: edema was observed until late time points (48 h p.i.), and the formation of large and purulent ulcerative abscesses were formed with the colonic submucosa, in association with the destruction of the tissue (Figure 5b). No signs of tissue destruction or inflammation were observed in the control animals (fed a high-ascorbate diet) using similar experimental procedures. In addition, we showed that *Shigella* could reach the blood circulation in ascorbate-deficient animals (oral and intrarectal challenge) but not in control animals (Figure 5d). *Shigella* colonized deep colonic layers, such as the submuscularis mucosa and mucosa, in ascorbate-deficient animals. Additionally, an important recruitment of neutrophils was observed within infected tissues 48 h p.i., with both challenges, while the colonization of the colonic mucosa by *Shigella* was more pronounced 24 h p.i. upon intrarectal challenge, although no changes in *Shigella* distribution was observed between challenges (Figure 6). Moreover, large populations of both *Shigella* and neutrophils were detected within the luminal compartment, as reported in patients with shigellosis, confirming the efficient dissemination of the bacteria during long-term infection. Conversely, in control animals, no tissue destruction was observed 24 h or 48 h p.i., with limited colonization of the mucosa by *Shigella* and only a few neutrophils were recruited (Figure S1).

Collectively, these findings confirmed that ascorbate deficiency increased the severity of shigellosis symptoms during extended periods of infection in guinea pigs. This infection model of shigellosis allowed for the first time to follow *Shigella* life cycle from oral challenge to the colonization and destruction of the colonic mucosa, despite innate immune response induction, as reported in humans. We further investigated the effect of ascorbate deficiency on *Shigella* infection susceptibility in a murine model.

### Invasion of colonic mucosa by Shigella is increased in young ascorbate-deficient Gulo^−/−^ mice

To evaluate the impact of ascorbate deficiency on the susceptibility of mice to *Shigella* infection, Gulo^−/−^ mice were used.^30^ First, ascorbate deficiency was induced in mice just after weaning (3-weeks old) by adding 0.01% ascorbate to drinking water for 5 weeks; 0.4% ascorbate corresponded to the control condition (Figure S2(A)). Ascorbate deficiency was confirmed by analyzing plasma ascorbate using HPLC (Figure S2(B)); however, no scurvy symptoms were observed. *S. flexneri* 2a infection was performed in 8-week mice by oral or intrarectal administration, after disruption of the microbiota with ampicillin treatment, because it stabilized the *Shigella* population in stools for one week after infection (Figure S2(C)). Although mice lost approximately 10% of their initial weight at 24 h p.i., they recovered at 3 days p.i. (Figure S2(D)). In contrast to ascorbate-deficient guinea pigs, ascorbate-deficient 8-week mice had no visible signs of shigellosis, and no bacterial colonization of the colonic mucosa was observed.

**Figure 7.**
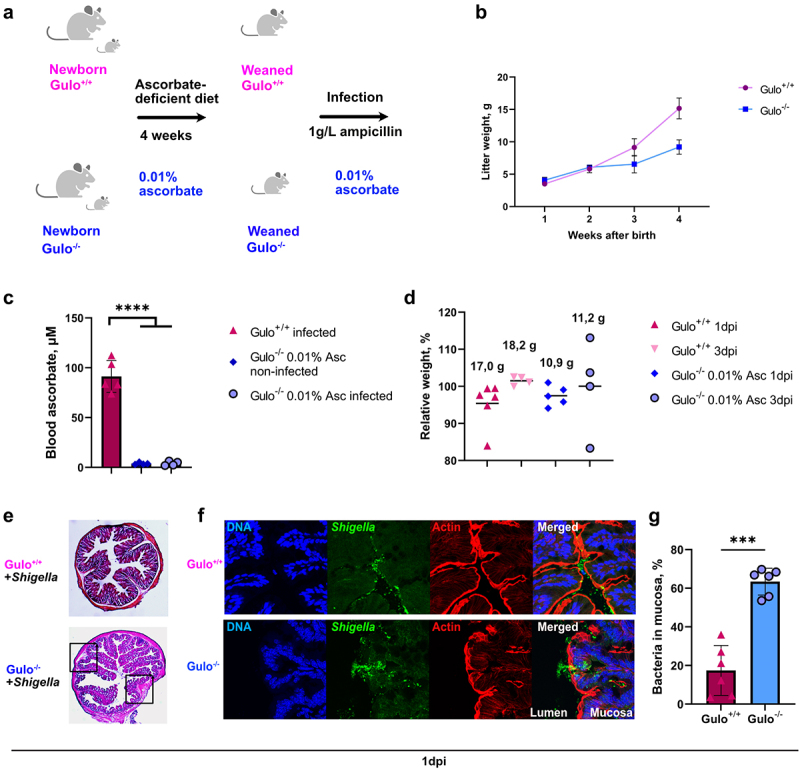
Early ascorbate deficiency induction in young mice leads to increased colon colonization by *Shigella* upon intrarectal administration. (a) Lactating mothers Gulo^−/−^ mice with new-born litters were treated with water containing 0.01% ascorbate after the day of birth. Mice were weaned 4 weeks later. Three days before and during infection all mice received water containing 0.01% ascorbate and 1 g/L ampicillin. (b) the weights of Gulo^−/−^ and Gulo^+/+^ mice were measured each week after birth. Dots represent means ± S.D. (c) Ascorbate concentration in mice plasma before and after *Shigella* infection was measured by HPLC. Columns indicate means ± S.E. Each dot represents one mouse. Significance was calculated by one-way ANOVA, ****p < .0001. (d) Mice relative weight loss was compared to the initial weight (100%) one and three days after infection (1 dpi and 3 dpi). Each dot represents an individual mouse, black solid lines demonstrate means. Average weights of mice in the groups indicated above the columns. (e) HE staining of colons of Gulo^+/+^ and Gulo^−/−^ mice 24 h after infection by *S. flexneri* 2a. Black squares indicate inflamed areas in colon from Gulo^−/−^ mice. (f) Representative pictures of murine colon mucosa colonization 24 h after infection by *S. flexneri* 2a in 4 weeks-old ascorbate-deficient Gulo^−/−^ and Gulo^+/+^ mice was visualized by immunofluorescent staining with DAPI for DNA (blue), anti-LPS serum for *S. flexneri* 2a (green) and phalloidin for actin (red). (g) Mucosa infection by *S. flexneri* 2a in Gulo^−/−^ mice was compared to Gulo^+/+^ mice. Individual bacteria were counted in the lumen and mucosa. Percentages of penetrated bacteria compared to total count of bacteria are represented as means ± S.D., *** indicates t-test *p* < .001.

Previously, it was reported that younger mice were more susceptible to *Shigella* infection.^13^ Thus, ascorbate deficiency was further induced just after birth for four weeks (Figure 7a). No scurvy symptoms were observed in young Gulo^−/−^ mice; however, significant mouse growth retardation was observed compared to the same age in Gulo^+/+^ mice (Figure 7b). Plasma ascorbate levels in Gulo^−/−^ mice fed a low-ascorbate diet were significantly lower than those in Gulo^+/+^ mice (Figure 7c). In these young ascorbate-deficient mice, no difference in relative weight loss upon *S. flexneri* 2a infection or visible signs of shigellosis were observed (Figure 7d). However, inflammation sites were observed in the colonic submucosal compartments of Gulo^−/−^ mice (Figure 7e). Increased *S. flexneri* 2a penetration into the colon mucosa was confirmed in ascorbate-deficient Gulo^−/−^ mice compared to Gulo^+/+^ mice (Figure 7f,g). Although these results confirmed that ascorbate deficiency increased the susceptibility of young mice to *Shigella* infection, additional specific defense mechanisms seemed to limit *Shigella* dissemination in this animal model.

## Discussion

While severe ascorbate deficiency is known to cause scurvy, we demonstrated here that moderate ascorbate deficiency is an important risk factor for shigellosis, which has so far been largely neglected and underestimated. This new insight raises the question of the definition of moderate ascorbate deficiency, which is currently not well characterized in humans, probably due to the difficulty in routinely administering plasma ascorbate. In guinea pigs, our results show that plasma ascorbate concentrations from 1.6 ± 0.3 µM to 3.5 ± 1.3 µM (vs 36.4 µM in animals fed with a high-ascorbate diet) are associated with increased shigellosis symptom severity. Further studies are required to define the upper values of moderate ascorbate deficiency defined in this study. In humans, an adequate ascorbate plasma concentration is estimated around 50 µM (confirmed in our previous work (49.5 ± 14.2 µM),^26^ while plasma ascorbate concentrations associated with insufficiency, moderate deficiency and severe deficiency are currently based on definitions rather than on clinical manifestations.^31^ Determining the relationship between ascorbate status and shigellosis development and severity requires further clinical studies, especially in children under the age of 5, in developing countries. In a previous study, it was reported that 20% of children in Puerto Rico were moderately deficient (<30 μM), with no correlation with diarrhea episodes.^32^ However, recent data from Central Africa obtained by our group demonstrated that infants with hypovitaminosis C were at a significantly higher risk of having a positive stool culture for various pathogenic bacteria including *Shigella*.^21^ Conversely, further investigations are required to evaluate the impact of shigellosis and other enteric diseases on ascorbate uptake and transport systems.

Vitamin C deficiency has been studied intensively in guinea pigs, but not in relation to shigellosis.^33,34^ In the present study, we validated a new animal model of shigellosis, the ascorbate-deficient guinea pig, which allowed us to follow for the first time the overall *Shigella* life cycle from an oral challenge to the colonization and destruction of the colonic mucosa. Until now, only transient infections have been observed, associated with foci of infection formation within the upper part of the colonic submucosa, *Shigella* being further cleared during the immune response in this model.^24,35^ The ascorbate-deficient guinea pig model of shigellosis can be a useful tool for the investigation of the immune response and dissemination of *Shigella* within the colonic mucosa, like we demonstrated with the influx of neutrophils into infection site. In addition, this model can be used to better understand the molecular bases of the subversion and antimicrobial activity of *Shigella* mediating its efficient dissemination within the infected tissues and to the bloodstream, as reported here. More specifically, this new shigellosis model will allow us to take a fresh look at the role of mucosal IgA in preventing *Shigella* dissemination^36,37^ and at *Shigella* capacity to subvert immune cell function and antimicrobial activity, which has been described previously *in vitro* or *in vivo* in other models (macrophage and B-lymphocyte apoptosis induction^38,39^ or T-cell migration.^40,41^ Most of these subversion mechanisms are dependent on *Shigella* Type Three Secretion System (T33S). As we have shown in a previous study that this secretion apparatus is mainly inactive within hypoxic foci of infection,^24^ alternative virulence mechanisms are expected to be involved, and may be identified in this new model of shigellosis. In addition, the ascorbate-deficient guinea pig model of shigellosis described in this study will allow investigation of the composition, structural organization, and role of isolated lymphoid follicles (belonging to the Gut-associated Lymphoid Tissue (GALT), specifically disseminated within the colonic mucosa) during *Shigella* infection, which has not yet been characterized.

In addition to the guinea pig infection model, we tested whether ascorbate-deficient Gulo^−/−^ mice could also be susceptible to *Shigella* colonization. Although we did not observe any shigellosis symptoms in the low-ascorbate murine model, we confirmed that ascorbate deficiency at the early stages of mouse development could determine more effective bacterial penetration into the colonic mucosa. Since vitamin C plays a key role in collagen production, higher colonization rates could be explained by reduced collagen production in 0.01% ascorbate mice, leading to more pervious intestines for *Shigella* infection. Several recent studies have demonstrated important differences in the cellular response to *Shigella* infection comparing murine and human cells.^12,42^ These studies showed that cellular responses associated with human-specific pyroptosis are important factors related to *Shigella*-sensitivity. Moreover, *Shigella* can specifically target gasdermin D and prevent pyroptosis in humans but not in murine cells.^42^ This could explain why we did no marked shigellosis symptoms were observed in mice even under high bacterial colonization.

Defining the detailed mechanisms involved in the increased susceptibility of ascorbate-deficient animals to *Shigella* infection requires further investigations. It has been previously shown that infection by enteropathogenic *E*. *coli* dysregulates the expression of L-ascorbic acid transporter SVCT1 and SVCT2.^43^ Low amount of ascorbate may impair the protective properties of epithelium barrier and tissue repair, while more global effects are anticipated on the oxidative stress level and the immune system function, since ascorbate is very important for the activity of both innate and adaptive immune cells. These cells tend to accumulate intracellular ascorbate; lymphocytes, monocytes and neutrophils contain ∼3.5, ∼3 and ∼1.5 mM of ascorbate, respectively, compared to 50 µM in plasma.^44^ Ascorbate stimulates neutrophil migration toward infectious sites and promotes phagocytosis. Ascorbate also sustains the differentiation and maturation of both B- and T-lymphocytes, particularly NK cells.^45^ It was recently shown that ascorbate boost the immunogenic properties of dendritic cells, through the NF-κB-dependent increase of TNFβ production.^46^ Further investigations will be required to evaluate the impact of ascorbate deficiency on the function of immune cells during shigellosis. It would be additionally of a great interest to evaluate the impact of ascorbate supplementation on the course of the infection disease in ascorbate-deficient animals.

Overall, we drew attention to ascorbate deficiency as an important factor determining shigellosis in both young guinea pig and mouse infection models. We presented a new *Shigella* infection model in young low-ascorbate guinea pigs, which allows the analysis of the entire cycle of *Shigella* colonization. This new model may boost further research on *Shigella* virulence mechanisms and vaccine development. We also demonstrated that early ascorbate deficiency induction in mice sensitized the animals to infection. Low ascorbate guinea pigs and Gulo^−/−^ mouse infection models will allow analysis of how immune cells in the colonic mucosa react to infectious *Shigella in vivo*.

These results are in agreement with our recent study in a mother-infant cohort showing that infants with hypovitaminosis C are at significantly higher risk of having a positive stool bacterial culture.^21^ The fact that ascorbate deficiency in young animals and children is a risk factor for *Shigella* infection but not in adults could be related to processes of immunity development in new-borns that are not yet fully understood, raising the question of the importance of ascorbate supplementation in population suffering from endemic diarrheal diseases, especially during childhood.

## Materials and methods

### Bacteria

Unless otherwise noted, *S. flexneri* 5a (M90T, wt, and ∆*mxiD* mutant) and 2a strains were grown at 37°C in Tryptic Soy Broth (TSB) with shaking or TSB agar plates supplemented with 0.01% Congo Red (Sigma-Aldrich) and ampicillin (100 μg/ml) when bacteria were transformed with the pGFP plasmid. *S. sonnei* was acquired from the Institut Pasteur strain collection (CIP 106,347), a clinical isolate from a 1999 Paris infection.^47^ The strain was grown in TSB supplemented with ampicillin (100 μg/ml) to maintain the pMW211 plasmid.

### Ascorbate-deficient guinea pig model of shigellosis

Young conventional guinea pigs (3 weeks old; female; Dunkin – Hartley; <150 g) were used to study *Shigella* infection severity. Guinea pigs were fed for 15 days with a standard diet allowing for an optimal ascorbate supply (400 mg ascorbate/kg, Safe ref. 106), as previously described^48^ or an ascorbate-deficient diet specifically designed and produced by the Safe (0, 10 mg, or 50 mg ascorbate/kg). Ascorbate deficiency was assessed based on its plasma concentration (see below). Guinea pig infection with *Shigella* infection was achieved with *S. flexneri* 5a wild-type and ∆*mxiD* strains, *S. flexneri* 2a and *S. sonnei* wild-type strains was performed by intrarectal challenge^24^ or oral challenge of animals with 10^10^ CFU exponentially grown, as indicated. In each experiment, at least three animals were used. Infections proceeded for 8 h, 24 h, 30 h or 48 h, as indicated, before the animals were sacrificed. All experimental procedures were approved by the Institute of Pasteur Ethics Committee (auth. n°190127).

The distal colon was sectioned (7 cm) and either flash-frozen in liquid nitrogen for tissue ascorbate abundance quantification (imaging mass spectrometry, see below) or fixed in 4% PFA in 1× PBS for 1–2 h, and then incubated in 1× PBS/glycine (100 mM) for 30 min to quench the PFA (for tissue labeling and imaging). PFA-fixed tissues were immersed successively in 15% and 30% sucrose at 4°C overnight. Tissue transversal sections (1 cm) were embedded in Tissue-Tek O.C.T. compound (Sakura) using a flash-freeze protocol and frozen at − 80°C. For histological and standard immunofluorescence staining, 10-μm sections were obtained using a CM-3050 cryostat (Leica Biosystems).

To quantify the abundance of bacteria in the bloodstream, 100 µL blood samples were collected in the presence of EDTA and plated on GTCS + ampicillin agar plates. Bacteria were counted after overnight culturing at 37°C.

### Ascorbate-deficient mouse model of shigellosis

Mice were fed irradiated Safe A03-A04 granules containing no ascorbate. Late ascorbate deficiency was induced by adding 0.01% ascorbate to mice drinking water for 5 weeks starting with 3 weeks-old Gulo^−/−^ mice. Early ascorbate deficiency was induced by adding 0.01% ascorbate for 4 weeks, starting on the first day of birth, while litters were still together with their mother. No symptoms of scurvy were observed. Three days before infection, 1 g/L ampicillin was added to the mice’s drinking water. *Shigella* infection was performed in 8 weeks-old mice or young 4 weeks-old just weaned mice by oral or intrarectal administration with 10^9^ infection dose per mouse. A low-ascorbate diet was also administered during the infection period. The appearance and weight of the mice were monitored daily. Infections proceeded for 24 h or 72 h, as indicated, before the animals were sacrificed. The experimental procedures were authorized by the Ministry of Higher Education, Research, and Innovation Ethics Committee (auth. n°APAFIS#29175–2021010711306157v1). Experiments with mice were performed in animal facilities at the Chronobiotron, an animal facility part of the National Research Infrastructure CELPHEDIA (Creation, Breeding, Phenotyping, Distribution and Archiving of model organisms). Murine colons were treated and analyzed as indicated in the ascorbate-deficient guinea pig model of shigellosis.

### Plasma ascorbate dosage

Blood samples were collected via intracardiac puncture in the presence of EDTA. Following centrifugation for 5 min at 2,000 *× g*, plasma was acidified with an equal volume of 10% (w/v) metaphosphoric acid (MPA) containing 2 mmol/L of disodium-EDTA. Ascorbate concentration was quantified by high-performance liquid chromatography with coulometric detection, as described previously.^26^ Results were averaged from the dosage performed on at least four animals per condition.

### Tissue ascorbate quantification

Imaging mass spectrometry was performed in collaboration with the team of Axel Walch (Helmholtz Zentrum München) on flash-frozen colonic samples. Each indicated ascorbate quantification was averaged from three areas designed on three independent tissues (three animals per condition). Metabolite mapping was performed using frozen guinea pig colonic tissue samples (12 μm, Leica Microsystems, CM1950, Germany) mounted onto indium tin oxide (ITO)-coated glass slides (Bruker Daltonik, Bremen, Germany) pretreated with 1:1 poly-Lysine (Sigma Aldrich, Munich, Germany) and 0.1% Nonidet *P*-40 (Sigma). Air-dried tissue sections were spray-coated with 10 mg/ml 9-aminoacridine hydrochloride monohydrate matrix (Sigma-Aldrich, Munich, Germany) in 70% methanol using a SunCollect™ sprayer (Sunchrom, Friedrichsdorf, Germany). Spray-coating of the matrix was conducted in eight passes (ascending flow rates of 10 µL/min, 20 µL/min, and 30 µL/min for layers 1–3, and 40 µL/min for layers 4–8), utilizing a 2 mm line distance and a spray velocity of 900 mm/min. Metabolites were detected in the negative-ion mode on a 7T Solarix XR Fourier-transform ion cyclotron resonance (FTICR) mass spectrometer (Bruker Daltonik) equipped with a dual ESI-MALDI source and a smartbeam-II Nd:YAG (355 nm) laser. Data acquisition parameters were specified in ftmsControl software 2.2 and flexImaging (v. 5.0) (Bruker Daltonik). Mass spectra were acquired in negative-ion mode covering ⁄ 75–1100, with a 1 M transient (0.367 s duration) and an estimated resolving power of 49,000 at m/z 200,000. The laser was operated at a frequency of 1,000 Hz utilizing 200 laser shots per pixel with a pixel resolution of 15 μm. L-arginine was used for external calibration in the ESI mode. On-tissue MS/MS was conducted on guinea pig colonic tissue sections using continuous accumulation of the selected ion mode and collision-induced dissociation (CID) in the collision cell. MS/MS spectra were analyzed using Bruker Compass DataAnalysis 5.0 (Build 203.2.3586).

### Tissue labelling and imaging

For histological studies, sectioned colonic samples were stained with hematoxylin-eosin following standard procedures.^49^ Labeled tissues were imaged with a transmission light microscope (Axioskop 2, Zeiss) using x10 or x20 objectives. For immunofluorescence imaging, PFA-fixed sectioned tissues were washed thrice in PBS + 0.1% saponin prior to immunolabelling in the same buffer for 1 h. Nuclei were stained with DAPI (1 mg/mL, Life Technologies,1:1000), Alexa Fluor 555 Phalloidin (Invitrogen, 1:40) for actin, or Myelotracker-Cy3 (1 mg/mL, 1:1000), a specific neutrophil marker designed and validated in our laboratory.^22^ After three additional washes in PBS + 0.1% saponin, three times in PBS, three times in deionized H_2_0 coverslips were mounted with Prolong gold mounting media (Thermo Fisher Scientific). Immunolabelled guinea pig colons infected with *Shigella* spp. (pGFP-positive or stained with 1:500 dilution of anti-LPS serum from rabbits) strains were imaged using a laser-scanning TCS SP8 confocal microscope (Leica). Z-stack images were taken with 1 μM step size increments. The obtained Z-stack images were processed using Fiji software.

### Statistics

Data were analyzed using Prism 8 software (GraphPad). ANOVA or Student’s t-test were performed to analyze the different datasets.

## Supplementary Material

Supplemental Material

## Data Availability

The authors confirm that the data supporting the findings of this study are available within the article and its supplementary materials. The data supporting the findings of this study are available from the corresponding author, B.S.M., upon reasonable request.
