# Supplementary material for: Ascorbate deficiency increases progression of shigellosis in guinea pigs and mice infection models
Source: Gut Microbes. 2023 Oct 24;15(2):2271597. doi: 10.1080/19490976.2023.2271597 (PMC10730169; doi:10.1080/19490976.2023.2271597)
Supplement: Supplemental Material [file KGMI_A_2271597_SM1734.zip › KGMI_A_2271597-supplemental figures/Supplemental figures captions.docx]

Figure S1. *S. flexneri* 5a do not colonize control guinea pigs. *S. flexneri* 5a pGFP (green) and neutrophils (red) were detected by immunofluorescence within the distal colonic mucosa of control guinea pigs (400 mg ascorbate/kg diet) challenged orally or intrarectally for 24 and 48h with 10^10^ CFU. Nucleus was stained with DAPI (blue), neutrophils were stained with Myelotracker-Cy3 (red). Scale bars are 150 µm. Representative images are presented of at least 5 slices from 3 different animals.

Figure S2. Late ascorbate deficiency induction in 8 weeks old mice. (A) Weaned Gulo^-/-^ mice were treated with water containing 0.4% or 0.01% ascorbate for 5 weeks. Three days before and during infection mice received water containing 0.4% or 0.01% ascorbate and 1g/L ampicillin. C57BL/6 mice were given regular water. (B) Ascorbate concentrations in mice plasma before and after *Shigella* infection was measured by HPLC. Columns indicate means ± S.E. Each dot represents one mouse. Significance was calculated by one-way ANOVA, ****p<0.0001. (C) *Shigella* counts in infected mice stools. CFU (colony forming units) were counted on agar plates. Ampicillin was added three days before infection into mice drinking water. Trendlines are indicated by solid lines. (D) Mice relative weight loss was compared to the initial weight (100%) one and three days after infection (1 dpi and 3 dpi). Each dot represents an individual mouse, black solid lines demonstrate means. Average weights of mice in the groups indicated above the dots.
