# Supplementary figures and images for: Ascorbate deficiency increases progression of shigellosis in guinea pigs and mice infection models
Source: Gut Microbes. 2023 Oct 24;15(2):2271597. doi: 10.1080/19490976.2023.2271597 (PMC10730169; doi:10.1080/19490976.2023.2271597)

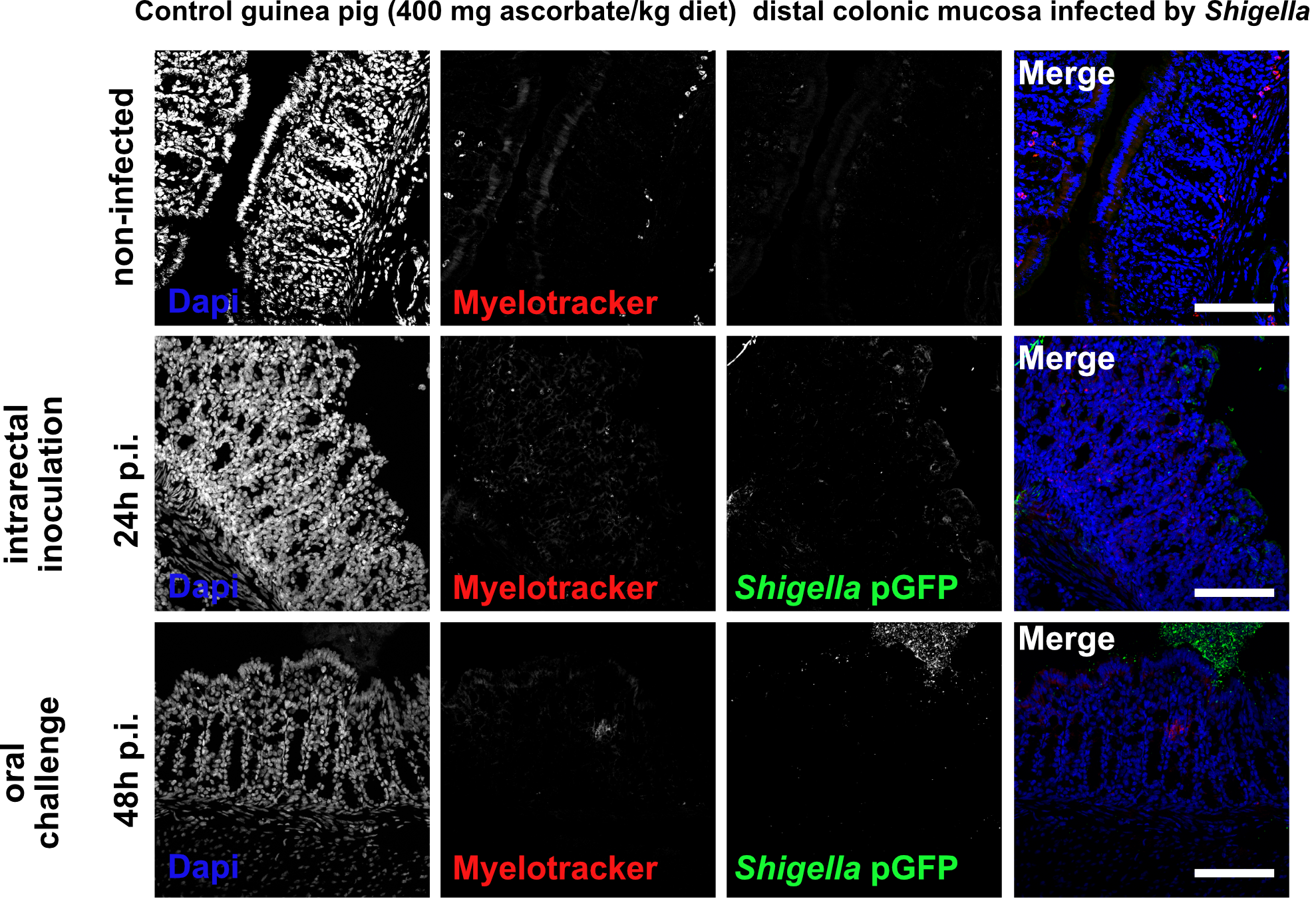

Supplement: Supplemental Material [file KGMI_A_2271597_SM1734.zip › KGMI_A_2271597-supplemental figures/Skerniskyte et al Figure S1.tiff]

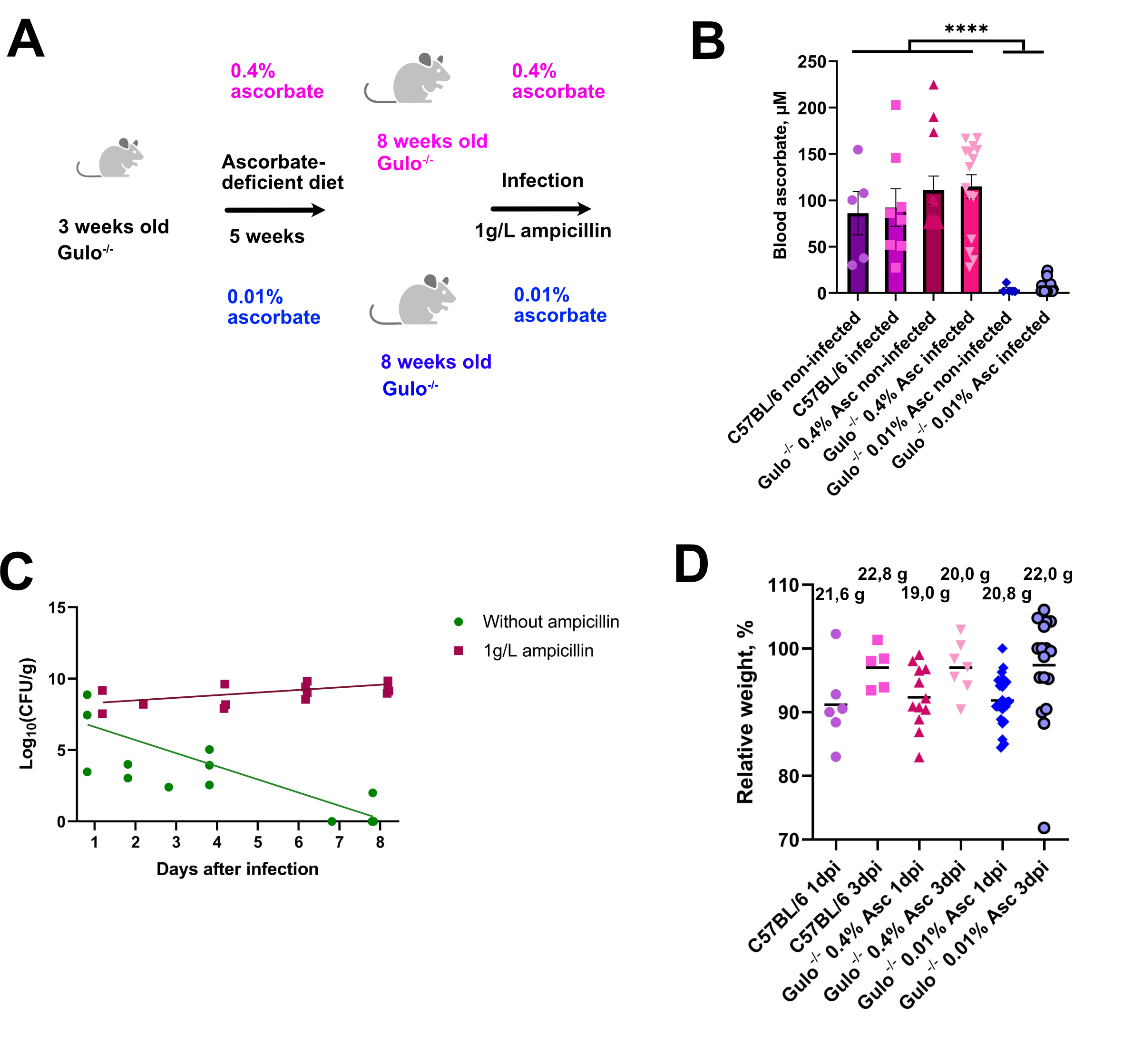

Supplement: Supplemental Material [file KGMI_A_2271597_SM1734.zip › KGMI_A_2271597-supplemental figures/Skerniskyte et al Figure S2.tiff]
